# Summary statistics of learning link changing neural representations to behavior

**Published:** 2025-07-14

**Authors:** Jacob A. Zavatone-Veth, Blake Bordelon, Cengiz Pehlevan

**Affiliations:** 1 Center for Brain Science, Harvard University, Cambridge, MA, USA; 2 Society of Fellows, Harvard University, Cambridge, MA, USA; 3 John A. Paulson School of Engineering and Applied Sciences, Harvard University, Cambridge, MA, USA; 4 Kempner Institute for the Study of Natural and Artificial Intelligence, Harvard University, Cambridge, MA, USA

## Abstract

How can we make sense of large-scale recordings of neural activity across learning? Theories of neural network learning with their origins in statistical physics offer a potential answer: for a given task, there are often a small set of summary statistics that are sufficient to predict performance as the network learns. Here, we review recent advances in how summary statistics can be used to build theoretical understanding of neural network learning. We then argue for how this perspective can inform the analysis of neural data, enabling better understanding of learning in biological and artificial neural networks.

## I. INTRODUCTION

Experience reshapes neural population activity, molding an animal’s representations of the world as it learns to perform new tasks. Thanks to advances in experimental technologies, it is just now becoming possible to measure changes in the activity of large neural populations across the course of learning (Fink et al., 2025; Kriegeskorte and Wei, 2021; Masset et al., 2022; Steinmetz et al., 2021; Sun et al., 2025; Vaidya et al., 2025; Zhong et al., 2025). However, with this new capability comes the challenge of identifying which features of high-dimensional activity patterns are meaningful for understanding learning. While analyses of representations have begun how to elucidate how learning reshapes the structure of activity, it is not in general clear whether these measurements are sufficient to understand how representational changes relate to behavior (Krakauer et al., 2017; Kriegeskorte et al., 2008; Kriegeskorte and Wei, 2021; Sucholutsky et al., 2024).

In this Perspective, we propose that the principled identification of **summary statistics of learning** offers a possible path forward. This framework is grounded in theories of the statistical physics of learning in neural networks, which show that low-dimensional summary statistics are often sufficient to predict task performance over the course of learning (Engel and van den Broeck, 2001; Watkin et al., 1993; Zdeborová and Krzakala, 2016). We argue that thinking systematically about summary statistics gives new insight into what existing approaches of quantifying neural representations reveal about learning, and allows identification of what additional measurements would be required to constrain models of plasticity. We emphasize that the goal of this Perspective is not to advocate for the use of a particular set of summary statistics, but rather to explain the general philosophy of this approach to understanding learning in high dimensions.

## II. WHAT IS A SUMMARY STATISTIC?

We posit that summary statistics of learning must satisfy two minimal desiderata:

1. **They must be low-dimensional.** That is, their dimension is low relative to the number of neurons in the network of interest. Indeed, most summary statistics we will encounter are determined by averages over the population of neurons.
2. **They must be sufficient to predict behavior across learning.** From a theoretical standpoint, there should exist a closed set of equations describing the evolution of the summary statistics that predict the network’s performance.

As we will illustrate with concrete examples in Section III, summary statistics satisfying these two desiderata are often highly interpretable thanks to their clear relationship to the network architecture and learning task. However, the summary statistics relevant for predicting performance may not be sufficient to predict all statistical properties of population activity. We will elaborate on this issue, and the resulting limitations of descriptions based on summary statistics alone, in Section IV.

Our use of the term “summary statistics” follows work by Ben Arous and colleagues (Ben Arous et al., 2023, 2022). In the literature on the statistical physics of learning, the quantities that we refer to as summary statistics are often termed “order parameters” (Engel and van den Broeck, 2001; Mézard et al., 1987; Watkin et al., 1993; Zdeborová and Krzakala, 2016). We prefer to use the former, more general term as it better captures the goal of these reduced descriptions in a neuroscientific context: we aim to summarize the features of neural activity relevant for learning.

## III. SUMMARY STATISTICS IN THEORIES OF NEURAL NETWORK LEARNING

We now review how summary statistics emerge naturally in theoretical analyses of neural network learning. Out of many theoretical results, we focus on two example settings: online learning from high-dimensional data in shallow networks, and batch learning in wide and deep networks (Arnaboldi et al., 2023; Ben Arous et al., 2023; Bordelon et al., 2025; Bordelon and Pehlevan, 2023b; Cui et al., 2023; Engel and van den Broeck, 2001; Goldt et al., 2019; van Meegen and Sompolinsky, 2025; Saad and Solla, 1995; Saxe et al., 2013; Watkin et al., 1993; Zavatone-Veth and Pehlevan, 2021; Zavatone-Veth et al., 2022b; Zdeborová and Krzakala, 2016). These model problems illustrate how relevant summary statistics may be identified given a task, network architecture, and learning rule.

### A. Online learning in shallow neural networks with high dimensional data

Classical models of online gradient descent learning in high dimensions can be often be summarized with simple summary statistics (Arnaboldi et al., 2023; Ben Arous et al., 2022; Biehl and Schwarze, 1995; Engel and van den Broeck, 2001; Goldt et al., 2019, 2020; Saad and Solla, 1995; Watkin et al., 1993). In this section, we discuss how the generalization performance of perceptrons and shallow (two-layer) neural networks trained on large quantities of high dimensional data can be summarized by simple weight alignment measures. Most simply, the perceptron model $f(x)=\sigma \left(\frac{1}{\sqrt{D}}w\cdot x\right)$ seeks to learn a weight vector $w\in R^{D}$ which correctly classifies a finite set of randomly sampled training input-output pairs ($x_{\mu }$, $y_{\mu }$). If the inputs are random, $x_{\mu }∼𝒩(0,I_{D})$, and the targets $y_{\mu }=y(x_{\mu })$ are generated by a **teacher network** $y(x)=\sigma \left(\frac{1}{\sqrt{D}}w_{⋆}\cdot x\right)$, then the generalization performance (performance of the model on new *unseen data*, $E_{x}[{\left(f(x)-y(x)\right)}^{2}]$ is completely determined by the overlap of w with itself and with the target direction $w_{⋆}$

$$Q=\frac{1}{D}w\cdot w\;,\;R=\frac{1}{D}w\cdot w_{⋆}.$$

If the learning rate is scaled appropriately with the dimension D, the high-dimensional (large-D) limit of online stochastic gradient descent is given by a deterministic set of equations for Q and R:

(1) $$\frac{d}{d\tau }\left[\begin{array}{c} Q(\tau )\\ R(\tau ) \end{array}\right]=F[Q(\tau ),R(\tau )],$$

where the continuous training ‘time’ τ is the ratio of the number of samples seen to the dimension and $F\;:R^{2}\to R^{2}$ is a nonlinear function that depends on the learning rate, the loss function, and the link function σ(⋅) (Arnaboldi et al., 2023; Ben Arous et al., 2022; Engel and van den Broeck, 2001; Goldt et al., 2019; Saad and Solla, 1995). Integrating this update equation allows one to predict the evolution of the generalization error as more training data are provided to the algorithm. Despite the infinite dimensionality of the original optimization problem, only two dimensions are necessary to capture the dynamics of generalization error.

The analysis of online perceptron learning can be extended to two layer neural networks with a small number of hidden neurons N,

$$f(x)=\frac{1}{N}\sum_{i=1}^{N}a_{i}\;\phi (h_{i}(x))\;\;h_{i}(x)=\frac{1}{\sqrt{D}}w_{i}\cdot x\;,\;i\in \{1,\ldots ,N\}.$$

$$y(x)=\sigma (h_{1}^{⋆}(x),\ldots ,h_{K}^{⋆}(x))\;\;\;h_{k}^{⋆}(x)=\frac{1}{\sqrt{D}}w_{k}^{⋆}\cdot x\;,\;k\in \{1,\ldots ,K\}.$$

In this setting with isotropic random data, the relevant summary statistics are the readout weights $a\in R^{N}$, along with matrices $Q\in R^{N\times N}$ and $R\in R^{N\times K}$ with entries

$$Q_{ij}=\frac{1}{D}w_{i}\cdot w_{j}\;,\;R_{ik}=\frac{1}{D}w_{i}\cdot w_{k}^{⋆}$$

For this system, we can track the gradient descent dynamics for a, Q, and R through a generalization of Equation (1) (Biehl and Schwarze, 1995; Goldt et al., 2019, 2020; Saad and Solla, 1995). This reduces the dimensionality of the dynamics from the N+DN trainable parameters {$a_{i}$}, {$w_{j}$} to $N+N^{2}+NK$ summary statistics, which is significant when D≫N+K. This reduction enables the application of analyses that cannot scale to high dimensions, for instance control-theoretic methods to study optimal learning hyperparameters and curricula (Mignacco and Mori, 2025; Mori et al., 2025). Recent works have also begun to study approximations to these summary statistics when the network width N is also large, as further dimensionality reduction if possible when Q and R have stereotyped structures (Arnaboldi et al., 2023; Montanari and Urbani, 2025).

Under what conditions is this reduction possible? Fundamentally, the summary statistics a, Q, and R are sufficient to determine the network’s performance so long as the preactivations $h_{i}$ and $h_{k}^{⋆}$ are approximately Gaussian. Thus, one can relax the assumption that the inputs x are exactly Gaussian so long as a central limit theorem applies to $h_{i}$ and $h_{k}^{⋆}$ (Goldt et al., 2019, 2020). Moreover, one can allow for correlations between the different input dimensions so long as $h_{i}$ and $h_{k}^{⋆}$ remain Gaussian. If $E[{\mathrm{xx}}^{T}]=\Sigma$, with a modification of the definition of the overlaps to $Q_{ij}=\frac{1}{D}w_{i}\cdot \Sigma w_{j}$ and $R_{ik}=\frac{1}{D}w_{i}\cdot \Sigma w_{k}^{⋆}$ a similar reduction applies (Arnaboldi et al., 2023). One can even consider extensions to plasticity rules other than stochastic gradient descent. For example, online node perturbation leads to a different effective dynamics for the same set of summary statistics (Hara et al., 2011, 2013).

How could the overlaps Q and R be accessed from measurements of neural activity? And, in the absence of detailed knowledge of a teacher network, how could one identify the relevant overlaps? Under the simple structural assumptions of these models, one could estimate the overlaps from covariances of network activity across stimuli, i.e., with isotropic inputs one has $E_{x}[h_{i}h_{k}^{⋆}]=R_{ik}$ and $E_{x}[h_{i}h_{j}]=Q_{ij}$. Moreover, one can in some cases detect this underlying low-dimensional structure by examining the principal components of the learning trajectory (Ben Arous et al., 2023). However, more theoretical work is required in this vein.

### B. Learning in wide and deep neural networks

**FIG. 1:**
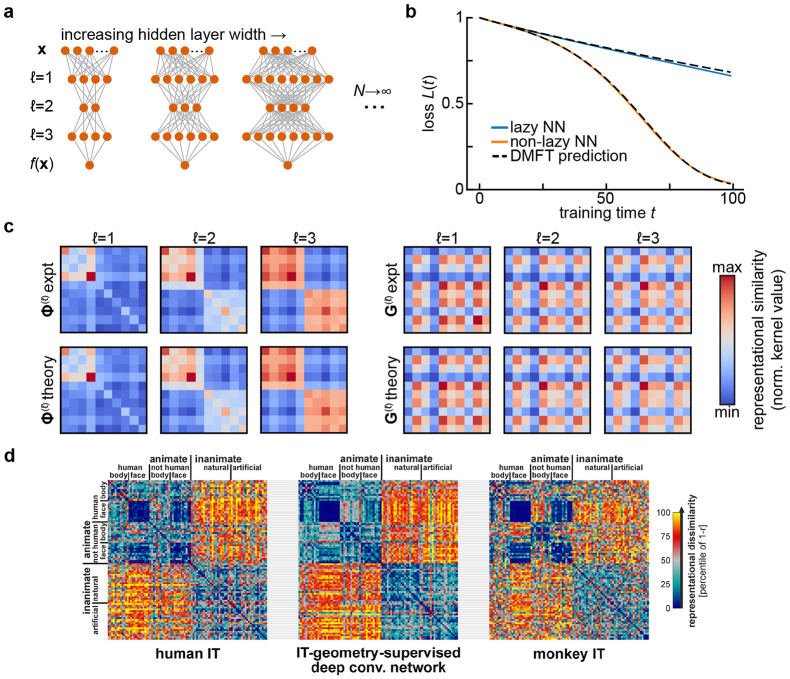
Representational similarity kernels in wide neural network models and in the brain. **a**. Diagram of the infinite-width limit of a deep feedforward neural network. For a fixed input and output dimension, one considers a sequence of networks of increasing hidden layer widths. **b**. Predicting the performance of width-2500 fully-connected networks with three hidden layers and tanh activations over training using the dynamical mean-field theory described in Section III. Networks are trained on a synthetic binary classification dataset of 10 examples, with 5 examples assigned each class at random. This leads to block structure in the final representations. Adapted from Bordelon and Pehlevan (2023b). **c**. The summary statistics in the dynamical mean field theory for the network in **b** are representational similarity kernels ($\Phi^{\left(\ell \right)}$; *left*) and gradient similarity kernels ($G^{\ell }$; *right*) for each layer. The top row shows kernels estimated from gradient descent training, and the bottom row the theoretical predictions. All kernels are shown at the end of training (t=100). Adapted from Bordelon and Pehlevan (2023b). **d**. Comparing representational similarity kernels across models and brains. Here, similarity is measured using the Pearson correlation r, and the *dissimilarity* 1−r is plotted as a heatmap. Kernels resulting from fMRI measurements of human inferior temporal (IT) cortex (*left*) and electrophysiological measurements of macaque monkey IT cortex (*right*) are compared with the kernel for features from a deep convolutional neural network after optimal re-weighting to match human IT (*center*). Adapted from Figure 10 of Khaligh-Razavi and Kriegeskorte (2014) with permission from N. Kriegeskorte under a CC-BY License.

Another strategy to reduce the complexity of multilayer deep neural networks is to analyze the dynamics of learning in terms of representational similarity matrices (kernels) for each hidden layer of the network. Consider, for example, a deep fully-connected network with input $x\in R^{D}$,

$$\;f(x,t)=\frac{1}{\gamma \sqrt{N}}\sum_{i=1}^{N}w_{i}(t)\phi (h_{i}^{\left(L\right)}(x,t)),\;h_{i}^{\left(\ell +1\right)}(x,t)=\frac{1}{\sqrt{N}}\sum_{j=1}^{N}W_{ij}^{\left(\ell \right)}(t)\phi (h_{j}^{\left(\ell \right)}(x,t)),\;\;\ell \in \{1,\ldots ,L+1\},\;\;h_{i}^{\left(1\right)}(x,t)=\frac{1}{\sqrt{D}}\sum_{j=1}^{D}W_{ij}^{\left(0\right)}(t)x_{j},$$

where t denotes training time. When the weights start from an uninformed initial condition (*i.e.*, Gaussian random matrices), and the network is optimized with full-batch gradient descent in the large width N→∞ limit (Figure 1), the relevant summary statistics of the model are **representational similarity matrices**

$$\Phi^{\left(\ell \right)}(x,x^{\prime },t,t^{\prime })=\frac{1}{N}\sum_{i=1}^{N}\phi (h_{i}^{\left(\ell \right)}(x,t))\phi (h_{i}^{\left(\ell \right)}(x^{\prime },t^{\prime }))$$

and gradient similarity matrices

$$G^{\left(\ell \right)}(x,x^{\prime },t,t^{\prime })=\frac{1}{N}\sum_{i=1}^{N}g_{i}^{\left(\ell \right)}(x,t)g_{i}^{\left(\ell \right)}(x^{\prime },t^{\prime }),\;\;g_{i}^{\left(\ell \right)}(x,t)\equiv \gamma \sqrt{N}\frac{\partial f(x,t)}{\partial h_{i}^{\left(\ell \right)}(x,t)},$$

which respectively compare the hidden states $\phi (h^{\left(\ell \right)}(x,t))$ and the gradient signals $g_{i}^{\left(\ell \right)}(x,t)$ at each hidden layer ℓ for each pair of data points (x, $x^{\prime }$) and each pair of training times (t, $t^{\prime }$). Depending on how weights and learning rates are scaled, one can obtain different types of large-width (N→∞) limits. In the *lazy / kernel* limit where γ is constant, these representational similarity matrices are static over the course of learning (Jacot et al., 2018). However, an alternative scaling ($\gamma \propto \sqrt{N}$) can be adopted where these objects evolve in a task-dependent manner even as N→∞ (Bordelon and Pehlevan, 2023b; Yang and Hu, 2021). In either case the performance of the network is determined by these objects since the outputs satisfy a differential equation

$$\frac{d}{dt}f(x,t)=-E_{x^{\prime }}\sum_{\ell }G^{\left(\ell +1\right)}(x,x^{\prime },t,t)\Phi^{\left(\ell \right)}(x,x^{\prime },t,t)\frac{\partial ℒ}{\partial f(x^{\prime },t)},$$

where ℒ is the loss function and $E_{x^{\prime }}$ denotes expectation over the training dataset. So long as the square of the number of training data points is small relative to the network width, this provides a substantial dimensionality reduction.

While this provides a description of the training dynamics of a model under gradient flow, it can be extended to other learning rules which use approximations of the backward pass variables ${\tilde{g}}_{i}^{\left(\ell \right)}(x,t)$, which we called pseudo-gradients in Bordelon and Pehlevan (2023a). Such rules include Hebbian learning, feedback alignment, and direct feedback alignment (Hebb, 2005; Lillicrap et al., 2016; Nøkland, 2016). In this case, the relevant summary statistics to characterize the prediction dynamics of the network include the gradient-pseudogradient correlation, which measures the alignment between the gradients used by the learning rule and the gradients that one would have used with gradient flow,

$${\tilde{G}}^{\left(\ell \right)}(x,x^{\prime },t,t^{\prime })=\frac{1}{N}\sum_{i=1}^{N}g_{i}^{\left(\ell \right)}(x,t){\tilde{g}}_{i}^{\left(\ell \right)}(x^{\prime },t^{\prime }),$$

as ${\tilde{G}}^{\left(\ell \right)}$ governs the evolution of the function output:

$$\frac{d}{dt}f(x,t)=-E_{x^{\prime }}\sum_{\ell }{\tilde{G}}^{\left(\ell +1\right)}(x,x^{\prime },t,t)\Phi^{\left(\ell \right)}(x,x^{\prime },t,t)\frac{\partial ℒ}{\partial f(x^{\prime },t)}.$$

A significant line of recent work in neuroscience aims to quantify neural representations and compare them across networks through analysis of representational similarity matrices $\Phi^{\left(\ell \right)}(x,x^{\prime })$ (Kriegeskorte et al., 2008; Sucholutsky et al., 2024; Williams, 2024; Williams et al., 2021). Here, we see that these kernel matrices arise naturally as summary statistics of forward signal propagation in wide and deep neural networks (Figure 1). At the same time, those results show that tracking only feature kernels is not in general sufficient to predict performance over the course of learning. One needs access also to coarse-grained information about the plasticity rule in the form of gradient kernels (either $G^{\left(\ell \right)}$ or ${\tilde{G}}^{\left(\ell \right)}$), and to information about the network outputs (for instance ∂ℒ∕∂f). More theoretical work is required to determine how to reliably estimate these gradient kernels from data.

## IV. IMPLICATIONS FOR NEURAL MEASUREMENTS

The two example settings detailed in Section III show how the relevant summary statistics of learning depend on network architecture and learning rule. Theoretical studies are just beginning to map out the full space of possible summary statistics for different network architectures (Arnaboldi et al., 2023; Ben Arous et al., 2023; Bordelon et al., 2025; Bordelon and Pehlevan, 2023b; Cui et al., 2023; Engel and van den Broeck, 2001; Goldt et al., 2019; van Meegen and Sompolinsky, 2025; Saad and Solla, 1995; Saxe et al., 2013; Zavatone-Veth and Pehlevan, 2021; Zavatone-Veth et al., 2022b; Zdeborová and Krzakala, 2016). Though details of the relevant summary statistics vary depending on the scaling regime and task—as illustrated by the examples above, where network width, training dataset size, and learning rule change the relevant statistics and their effective dynamics—they share broad structural principles. In all cases, summary statistics are defined by (weighted) averages over sub-populations of neurons within the network of interest, *e.g.*, correlations of activity with task-relevant variables, or autocorrelations of activity within a particular layer in a deep network. Thanks to these common structural features, these varied theories of summary statistics have common implications for the analysis and interpretation of neuroscience experiments.

### A. Benign subsampling

**FIG. 2:**
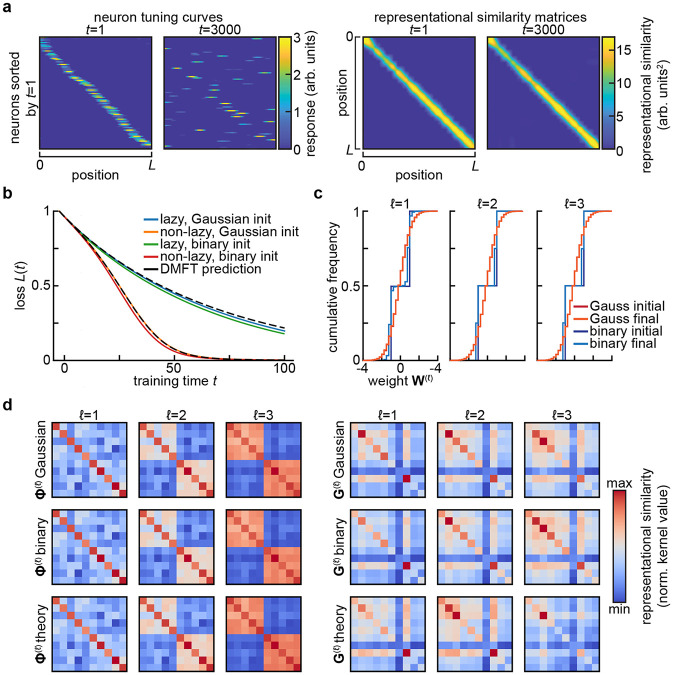
Invariance and universality in summary statistics. **a**. Stable summary statistics despite drifting single-neuron responses. In Qin et al. (2023)’s model of representational drift, single neurons are strongly tuned to a spatial variable, yet their tuning changes dramatically over time (*left*). Despite this drift, the similarity of the population representations of different spatial positions remains nearly constant (*right*). Adapted from Figure 5e of Qin et al. (2023), of which C.P. is the corresponding author. **b**. Universality of summary statistics in wide and deep networks with respect to the distribution of initial weights. Setting is as in Figure 1b-c, but also including a network for which the weights are initially drawn from {−1, +1} with equal probability. Here, N=2000, and a different realization of the random task is sampled relative to Figure 1b-c, so the loss curves are not identical. **c**. Cumulative distribution of weights at the start (*initial*) and end (*final*) of training for the networks shown in (**b**). Note that the small change in the weight distributions for the Gaussian-initialized networks is not visible at this resolution, and that one expects the size of weight changes to scale with $1∕\sqrt{N}$ (Bordelon and Pehlevan, 2023b). **d**. Feature and gradient kernels at the end of training for the networks in **b**. No substantial differences are visible between networks initialized with different weight distributions.

The summary statistics encountered in Section III are robust to subsampling thanks to their basic nature as averages over the population of neurons. These statistical theories in fact post a far stronger notion of benign subsampling: they result in neurons that are statistically exchangeable. This is highly advantageous from the perspective of long-term recordings of neural activity, as reliable measurement of summary statistics does not require one to track the exact same neurons over time. Instead, it suffices to measure a sufficiently large subpopulation on any given day. This obviates many of the challenges presented by tracking neurons over multiple recording sessions (Masset et al., 2022). Moreover, the variability and bias introduced by estimating summary statistics from a limited subset of relevant neurons can be characterized systematically (Bordelon and Pehlevan, 2024; Kang et al., 2025; Zavatone-Veth et al., 2022a). Taken together, these properties mean that summary statistics are relatively easy to estimate given limited neural measurements, provided that exchangability is not too strongly violated (Gao et al., 2017). We will return to this question in the Discussion, as a detailed analysis of the effects of non-identical neurons will be an important topic for future theoretical work. There are limits, however, to how far one can subsample. For instance, representational similarity kernels are more affected by small, coordinated changes in the tuning of many neurons than large changes in single-neuron tuning (Figure 2) (Kriegeskorte and Wei, 2021). Determining the minimum number of neurons one must record in order to predict generalization dynamics across learning will be an important subject for future theoretical work (Gao et al., 2017; Kriegeskorte and Wei, 2021).

### B. Invariances and representational drift

Though by our definition the summary statistics mentioned in Section III are sufficient to predict the network’s performance, they are not sufficient statistics for all properties of the neural code. In particular, in part because they arise from theories in which neurons become exchangable, they have many invariances. These invariances mean that individual tuning curves can change substantially without altering the population-level computation (Kriegeskorte and Wei, 2021). For instance, the representational similarity kernels are invariant under rotation of the neural code at each layer, enabling complete reorganization of the single-neuron code without any effect on behavior. Similarly, overlaps with task-relevant directions are invariant to changes in the null space of those low-dimensional projections. These invariances mean that focusing on summary statistics of learning sets a particular aperture on what aspects of representations one can assay.

At the same time, the invariances of summary statistics have important consequences for functional robustness. In particular, they are closely related to theories of representational drift, the seemingly puzzling phenomenon of continuing changes in neural representations of task-relevant variables despite stable behavioral performance (Masset et al., 2022; Rule et al., 2019). Many models of drift explicitly propose that representational changes are structured in such a way that certain summary statistics are preserved (Figure 2) (Masset et al., 2022; Pashakhanloo and Koulakov, 2023; Qin et al., 2023). Identifying the invariances of the summary statistics sufficient to determine task performance can allow for a more systematic characterization of what forms of drift can be accommodated by a given network. Conversely, identifying the invariances of a representation once task performance stabilizes might suggest which summary statistics are relevant for the learning problem at hand.

### C. Universality

An important lesson from the theory of high-dimensional statistics is that of *universality*: certain coarse-grained statistics are asymptotically insensitive to the details of the distribution. The most prominent example of statistical universality is the familiar central limit theorem: the distribution of the sample mean of independent random variables tends to a Gaussian as the number of samples becomes large. A broader class of universality principles arise in random matrix theory: the distribution of eigenvalues and eigenvectors of a random matrix often become insensitive to details of the distribution of the elements as the matrix becomes large. Most famously, the Marčenko-Pastur theorem specifies that the singular values of a matrix with independent elements have a distribution that depends only on the mean and variance of the elements (Marchenko and Pastur, 1967). In the context of learning problems, universality manifests through insensitivity of the model performance to details of the distributions of parameters or of features (Hu and Lu, 2022; Misiakiewicz and Saeed, 2024).

From the perspective of summary statistics, statistical universality can allow simple theories to make informative macroscopic predictions even if they do not capture detailed properties of single neurons. For instance, the mean-field description of the learning dynamics of wide neural networks introduced in Section III are universal in that they depend on the initial distribution of hidden layer weights only through its mean and variance, even though the details of that distribution will affect the distribution of weights throughout training (Figure 2) (Golikov and Yang, 2022; Williams, 1996). Like the invariances to transformations of the neural population code mentioned before, this is nonetheless a double-edged sword: these universality properties mean that focusing on predicting performance commits one to coarse-graining away certain microscopic aspects of neural activity. Though these features are not required to predict macroscopic behavior, they may be important for understanding biological mechanisms.

## V. DISCUSSION

The core insight of the statistical mechanics of learning in neural networks is the existence of low-dimensional summary statistics sufficient to predict behavioral performance. We have reviewed how different summary statistics emerge depending on network architecture and task, how summary statistics might be estimated from experimental recordings, and what this perspective reveals about existing approaches to quantifying representational changes over learning. We now conclude by discussing complementary summary statistics of neural representations that arise from alternative desiderata, and future directions for theoretical inquiry.

The summary statistics discussed here explicitly depend on the architecture and nature of plasticity in the neural network of interest, as they seek to predict its performance over learning. A distinct set of summary statistics arises if one aims to study what features of a representation are relevant for an *independently-trained* decoder. In this line of work, one regards the representation as fixed, rather than considering end-to-end training of the full network as we considered here. If the decoder is a simple linear regressor that predicts a continuous variable, the relevant summary statistics of the representation are just its mean and covariance across stimuli (Hu and Lu, 2022; Misiakiewicz and Saeed, 2024). Given a particular task, the covariance can be further distilled into the rate of decay of its eigenvalues and of the projections of the task direction into its eigenvectors (Atanasov et al., 2024; Bordelon et al., 2023; Bordelon and Pehlevan, 2022; Canatar et al., 2021, 2024; Harvey et al., 2024; Hastie et al., 2022; Williams, 2024). For categorically-structured stimuli, a substantial body of work has elucidated the summary statistics that emerge from assuming that one wants to divide the data according to a random dichotomy (Bernardi et al., 2020; Chung et al., 2018; Cohen et al., 2020; Engel and van den Broeck, 2001; Farrell et al., 2022; Harvey et al., 2024; Sorscher et al., 2022; Zavatone-Veth and Pehlevan, 2022).

The models reviewed here are composed of exchangable neurons, which simplifies the relevant summary statistics and renders them particularly robust to subsampling. However, the brain has rich structure that can affect which summary statistics are sufficient to track learning and how those summary statistics may be measured. Biological neural networks are embedded in space, and their connectivity and selectivity is shaped by spatial structure (Chklovskii et al., 2002; Khona et al., 2025; Stiso and Bassett, 2018). Notably, many sensory areas are topographically organized: neurons with similar response properties are spatially proximal (Kandler et al., 2009; Murthy, 2011). Moreover, neurons can be classified into genetically-identifiable cell types (Zhang et al., 2023), which may play distinct functional roles during learning (Fink et al., 2025; Hirokawa et al., 2019). Future theoretical work must contend with these biological complexities in order to determine the relevant summary statistics of learning subject to these constraints.
